# CCL4 and MIF: Prognostic Biomarkers for Evaluating the Chemoradiotherapy Response and Prognosis in Patients with ESCC

**DOI:** 10.7150/jca.104088

**Published:** 2025-03-03

**Authors:** Yuwen Wang, Chunxue Ding, Xiaoying Wei, Yuting Li, Xuyao Yu, Xi Chen, Jifeng Sun, Junhong Zhuang, Shuai Cao, Peng Zhen, Fang Fang, Jiarui Zhang, Jun Wang, Dong Qian, Qingsong Pang

**Affiliations:** 1 Department of Radiation Oncology, Tianjin Cancer Hospital Airport Hospital, Tianjin Medical University Cancer Institute and Hospital, National Clinical Research Center for Cancer, Tianjin's Clinical Research Center for Cancer, Key Laboratory of Cancer Prevention and Therapy, Tianjin, China; 2 Department of Radiation Oncology, the First Affiliated Hospital of USTC, Division of Life Sciences and Medicine, University of Science and Technology of China, Hefei, Anhui, 230001, China.; 3 Department of Radiation Oncology, Department of Pathology, Life Science Research Center, The First Affiliated Hospital of Xinxiang Medical University, China.; 4 Department of Radiation Oncology, Chifeng Tumor Hospital, Chifeng, China.

**Keywords:** ESCC, CCL4, MIF, chemoradiotherapy, immune infiltration

## Abstract

**Background**: Radiotherapy plays a central role in therapeutic strategy of local-advanced oesophageal squamous cell carcinoma. The aim of this study was to investigate cytokine profiles in serum of patients with ESCC and evaluate the potential utility of cytokine markers in predicting CRT response and prognostic prediction.

**Methods:** CCL4, MIF and CXCL8 in the serum samples who were participating in a phase II clinical trial (NCT02959385) were determined. The association between these cytokines and CRT response as well as prognostic prediction were subsequently assessed in ESCC. Subsequently, the results were verified in ESCC tissue and in the Cancer Genome Atals (TCGA) database.

**Results:** The expression of 120 cytokines were evaluated in serum of 4 ESCC patients with excellent CRT-response and 4 patients with CRT-resistance by cytokine microarrays. CCL3, CCL4, MIF, PLAUR and CXCL8 were screened. CCL4, MIF and CXCL8 were further detected by ELISA in other 60 patients enrolled in this study. Upregulation of CCL4, CXCL8 and MIF were observed in patients with excellent CRT-response. Elevated expression of CCL4 and MIF were closely associated with improved PFS and OS outcomes. Similar results were obtained in other 46 ESCC tumor tissues. 82 ESCC patients in TCGA database with increased CCL4 and MIF expression exhibited the favorable immunocyte infiltration and enriched immune response-related pathways, which indicates the preferable tumor immunogenicity.

**Conclusions:** CCL4 and MIF are identified as dependable and prognostic biomarkers for evaluating the response to CRT and prognosis in patients with ESCC.

## Introduction

Esophageal cancer remains a major health concern worldwide as it is the eighth most common cancer and the sixth leading cause of cancer-related mortality. Esophageal squamous cell carcinoma (ESCC) accounts for most esophageal carcinomas, which accounts for more than half of the global burden, with a 5-year overall survival rate of only 15-25% 1-3. Radiotherapy (IR) plays a central role in therapeutic strategy of local-advanced ESCC. However, the clinical response of ESCC to radiotherapy is not well predicted by existing diagnostic modalities 4, 5. Therefore, it is highly desirable to identify reliable and novel factors to predict radiosensitivity and improve patient outcomes.

Cytokines are highly inducible, soluble cell-signaling proteins with low molecular weights that function as intercellular mediators, set cell growth processes, and participate in cell differentiation, migration, and apoptosis 6. Cytokines play a key role in controlling communication between cells in the tumor microenvironment. Under certain circumstances, cytokines may orchestrate the host immune response against tumor cells; however, there is currently an expanding body of evidence linking cytokine networks to tumor growth, progression, metastasis, and host immunosuppression 6-8. These findings led to the hypothesis that alterations in cytokine levels in the ESCC microenvironment may have clinical significance in cancer development. Previous studies have implicated cytokines in tumor proliferation, angiogenesis, and metastasis in ESCC and have presented them as prognostic factors 9. Cytokines are secreted when subjected to external stimulation; however, the details of cytokine expression in response to IR and their significance in ESCC prognosis and therapeutic response remain unclear.

In the present prospective study, we performed 120 known tumor-related cytokine expression analyses in the pretreatment serum of patients with ESCC with different chemoradiotherapy (CRT) responses using a cytokine microarray. Among these, CCL4, CXCL8, and MIF levels were significantly higher in the serum of patients with excellent CRT-response response than in those with CRT resistance. CCL4, CXCL8, and MIF have been shown to induce angiogenesis and immune escape of tumor cells and promote the progression of many human cancers 10-13. CCL4, CXCL8, and MIF are cytokines with interacting signaling pathways. In this study, the expression of CCL4, CXCL8, and MIF in the serum were detected in a set of 68 patients with ESCC, and the predictive value of CCL4, CXCL8, and MIF for patients' response and survival were also investigated.

## Materials and Methods

### Patients

Sixty-eight patients for hematology research and other forty-eight patients for histology research with histologically confirmed ESCC treated between May 2015 and October 2016 at Tianjin Medical University Cancer Institute and Hospital were enrolled in this study. None of the one hundred and sixteen patients received anticancer treatment in a prehospital setting. All patients were recruited in a clinical trial (NCT02959385) and received CRT with cisplatin-based chemotherapy and concomitant radiotherapy (daily dose of 2.0 Gy for a total of 40-60 Gy over 4-6 weeks). Informed consent was obtained from each patient for blood sample collection during pre-treatment. All patients were staged according to the 8th edition of the American Joint Committee on Cancer Staging Manual: esophagus and esophogastric junction. This study was approved by the medical ethics committee of our institute.

When patients completed treatment at a total dose of 40 Gy of radiotherapy, the CRT response was evaluated clinically for primary lesions based on esophagography, CT, and endoscopic ultrasonography according to the World Health Organization criteria. The evaluation criteria for curative effects were complete response (CR), partial response (PR), stable disease (SD), and progressive disease (PD). Most patients underwent follow-up examinations at Tianjin Medical University Cancer Institute and Hospital.

### Serum samples collection and cytokine detection

Blood samples were collected during pretreatment. Venous blood samples were collected into serum tubes and allowed to clot. After centrifugation, serum samples were collected, aliquoted, and stored at -80 ℃ until analysis. Simultaneous detection of multiple cytokines provides a powerful tool for studying these cytokines. We detected serum concentrations of 120 known tumor-related cytokines by the RayBio® G-Series Cytokine Antibody Array, G-Series 1000, which is a glass slide that is a highly sensitive approach to simultaneously detect multiple cytokine expression levels from diverse sample types.

### Tissue samples collection and immunohistochemistry (IHC)

Tissue samples were collected by endoscopic biopsy during pretreatment. Fresh tissue specimens were stored at -80 ℃ until analysis. The specimens were formalin-fixed, paraffin-embedded, and sectioned. The expression levels of CCL4, MIF, and CXCL8 in tumor tissues were detected using IHC in the remaining 46 patients enrolled in this study.

### Immune infiltration and gene set enrichment analysis (GSEA)

Based on the CIBERSORT algorithm, the infiltration abundances of 22 distinct immunocyte subtypes in distinct expression subgroups were evaluated. The R limma package was used to perform whole-genome differential expression analysis, and the t-values derived from the differential results were regarded as input variables for GSEA, which was embedded in the R fgsea package. The Signaling pathways stored in the KEGG database were used for annotation comparison. Eighty-two ESCC patients in TCGA database were enrolled in this study.

### Statistical analysis

Statistical analysis was performed using R software (version 4.2.1). The Wilcoxon rank-sum test was used to explore the association between distinct response subgroups and cytokine expression. The chi-squared test was used to analyze the association between the expression of the three factors and the clinicopathological features of patients with ESCC. Receiver operating characteristic (ROC) curve analysis was performed to determine cutoff values using the R pROC package. The Kaplan-Meier survival method was used to obtain survival curves, and the log-rank test was used to compare differences. Multivariate Cox regression analyses with multiple confounding factors taken into account were performed using the R package forest model. Differences were considered significant when the *P* values were <0.05.

## Results

### Screening of serum cytokines related to radiosensitivity in patients with ESCC

**Figure 1:**
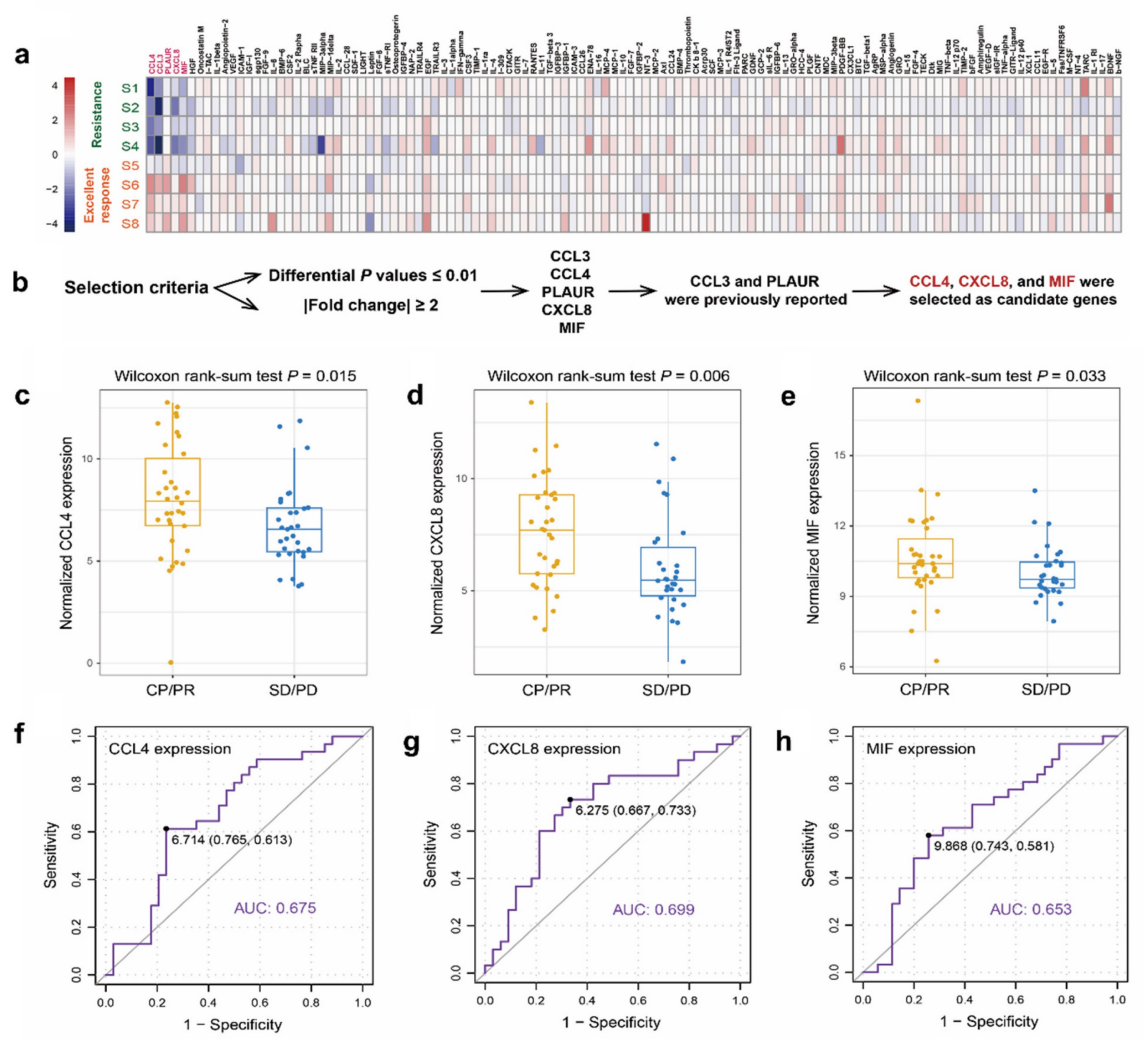
** Screening of serum cytokines related to radiosensitivity in patients with ESCC.** a. 120 known tumor-related cytokines expression analyses were performed in pretherapy serum from four patients with excellent CRT response (CR during CRT) and four patients with CRT-resistance (PD during CRT). b. Five differentially expressed cytokines were screened out by cytokine antibody arrays by differential expression analysis. c-e. Wilcoxon rank sum test revealed a promising predictive value of CCL8 (c), CXCL8 (d), and MIF (e) regarding CRT response in patients with ESCC (P = 0.015; *P* = 0.006; *P* = 0.033, respectively). f-h. ROC curve revealed a promising predictive value of CCL4 (f), CXCL8 (g), and MIF (h) regarding CRT response (AUC=0.675; AUC=0.699; AUC=0.653, respectively). Next, the cutoff scores for the high expression of CCL4 (f), CXCL8 (g), and MIF (h) were defined as more than 6.714 pg/mL, 6.275 pg/mL, and 9.868 pg/mL, respectively.

As shown in Figure 1a, a cytokine microarray containing 120 human cytokines was performed to compare the cytokine expression profiles of four patients with excellent CRT response (CR during CRT) and four patients with CRT resistance (PD during CRT). Differential expression analysis (supplementary table S1) showed that five differentially expressed cytokines (CCL3, CCL4, PLAUR, CXCL8, and MIF) were detected in patients with excellent CRT response compared to CRT-resistant patients (Figure 1a) under specific selection criteria (Figure 1b). CCL4, CXCL8, and MIF, which showed significantly high expression in patients with good CRT response (Figure 1c, d, e), were chosen for further study in an additional 60 patients, which were consistent with the microarray results.

### Identification of expression pattern of serum CCL4, CXCL8, and MIF

CCL4, CXCL8, and MIF showed significantly high expression in patients with ESCC with a good CRT response. The median pre-therapy serum MIF, IL-8, and MIP-1β concentrations were 4426.13 pg/mL (76.21-165296.60), 537.72 pg/mL (3.57-10759.83), and 754.84 pg/mL (1.03-6944.77), respectively. As the 95% confidence spatial data were very different, they were normalized. To identify a reasonable cutoff value for tumor “high expression,” ROC curve analysis was performed using the 0,1-criterion, which maximizes both sensitivity and specificity for the outcome 14. Herein, we analyzed the expression of CCL4, CXCL8, and MIF using ROC curve analysis with the CRT response. Our results demonstrated the promising predictive value of CCL4, CXCL8, and MIF for the CRT response in patients with ESCC (CCL4, AUC=0.675, Figure 1f; CXCL8, AUC=0.699, Figure 1g; MIF, AUC=0.653, Figure 1h).

### Clinical pathological correlation of CCL4, CXCL8, and MIF for patients with ESCC

Of the 68 patients, 2 patients were excluded because of a lack of follow-up. Serum MIF expression analyses was performed in 66 patients, and serum CCL4 and CXCL8 expression analysis were performed in 65 and 63 patients, respectively (1 patient and 3 patients had insufficient serum samples, respectively). Table 1 summarizes the relationship between serum levels of the three cytokines and their clinicopathological features. No significant correlation was found between serum CCL4, CXCL8, or MIF expression and sex, age, smoking, operation, TNM stage, multifocal lesions, or tumor location. However, further analysis showed that serum CCL4, CXCL8, and MIF expression levels were significantly associated with CRT response. Higher levels of the three cytokines were observed more frequently in the CR/PR group than in the SD/PD group (*P*=0.002, 0.008, and 0.001, respectively; Table 1). Moreover, using the Wilcoxon rank-sum test, the upregulation of CCL4, CXCL8, and MIF were frequently detected in patients with excellent CRT-response (*P* = 0.015, Figure 1c; *P* = 0.006, Figure 1d; *P* = 0.033, Figure 1e, respectively).

### The CRT prognostic capacity of CCL4, CXCL8, and MIF for patients with ESCC

**Figure 2:**
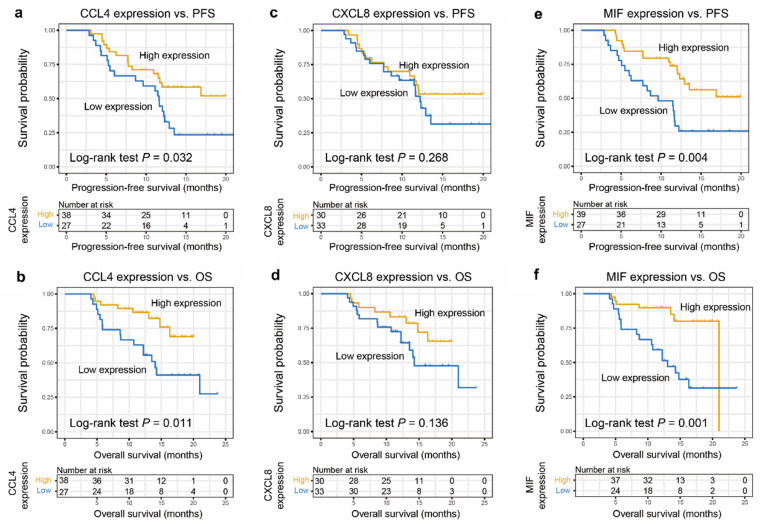
**Predictive significance of CCL4, CXCL8, and MIF for CRT response of patients with ESCC.** a, c, e. Higher levels of CCL4 (a), CXCL8 (c), and MIF (e) were associated with better PFS of patients with ESCC. Kaplan-Meier plots showing the progression-free survival curves of patients with ESCC according to CCL4, CXCL8, and MIF expression levels in serum (*P* = 0.032, 0.268, and 0.004, respectively). b, d, f. Kaplan-Meier plots showing the OS of patients with ESCC according to CCL4 (b), CXCL8 (d), and MIF (f) expression levels in serum (*P* = 0.011, 0.136 and 0.001, respectively).

**Figure 3:**
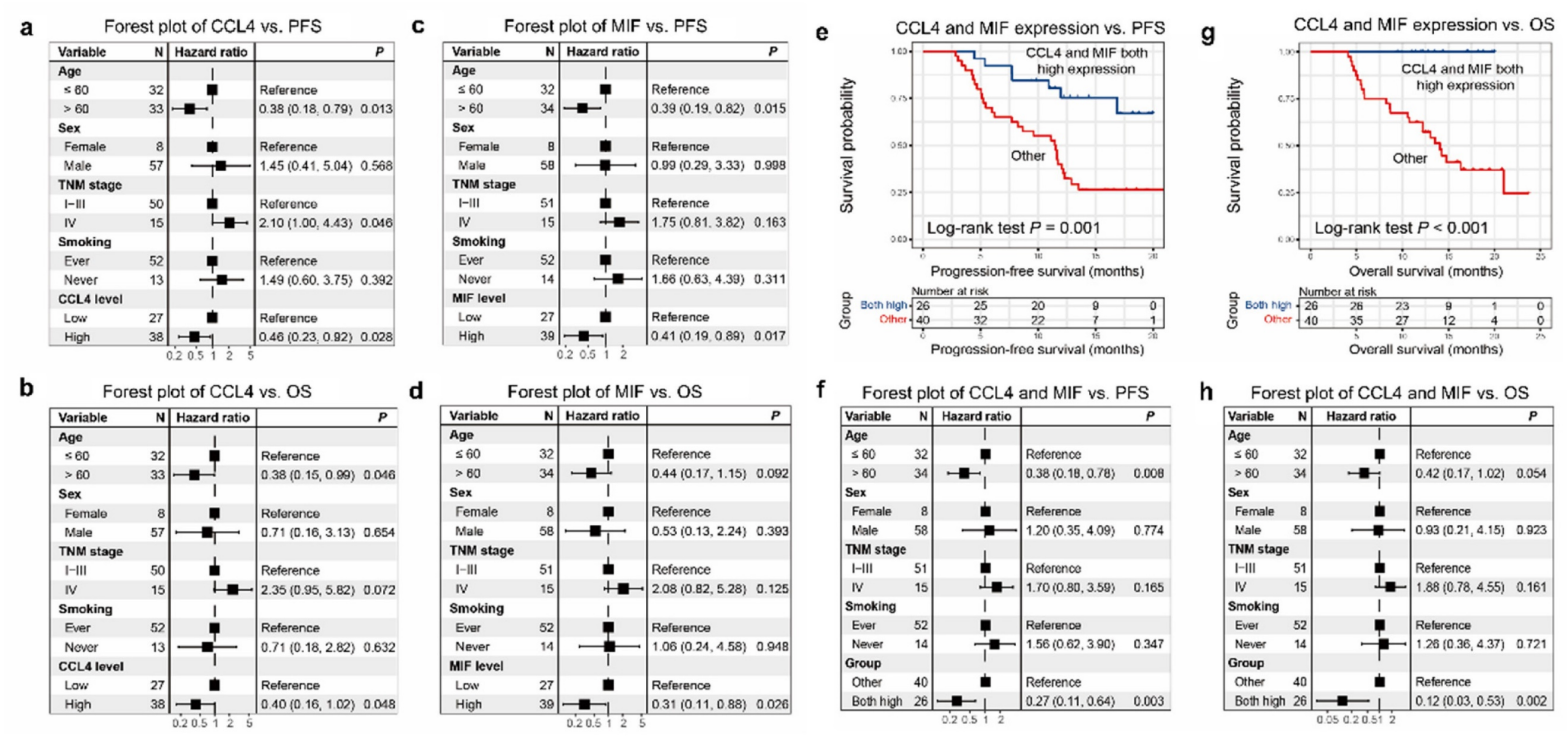
** Association between the expression of CCL4, CXCL8, and MIF and patients' survival demonstrated using forest plot analysis.** a-b. The forest plot analysis showed that CCL4 expression levels were independent predictors of PFS and OS (*P* = 0.028, Figure 4a;* P* = 0.048, Figure 4b, respectively). c-d. The forest plot analysis showed that MIF expression levels were independent predictors of PFS and OS (*P* = 0.017, Figure 4c;* P* = 0.026, Figure 4d, respectively). We designated the low-risk group as high expression of the two cytokines, and the high-risk group as other expression (regardless of their identity). e. The low-risk group was associated with better PFS in patients with ESCC (P=0.001). g. The low-risk group was associated with better OS of patients with ESCC (P<0.001). f. The forest plot analysis revealed a low-risk group was independent predictors of PFS (P=0.003). h. The forest plot analysis revealed that the low-risk group was an independent predictor of OS (P=0.002).

Higher CCL4 level was correlated closely with a greater progression-free survival (PFS) (*P*= 0.032), respectively (Figure 2a). High MIF level was positively correlated with PFS (*P*= 0.004; Figure 2e). Similarly, higher CCL4 and MIF levels were closely correlated with greater overall survival (OS) (*P* = 0.011, Figure 2b; *P* = 0.001, Figure 2f, respectively). However, higher CXCL8 level did not correlate with greater PFS or OS (*P* = 0.268, Figure 2c; *P* = 0.136, Figure 2d, respectively), although a tendency toward a favorable prognosis was observed. Subsequently, age, sex, TNM stage, smoking, and CCL4 or MIF expression were included in a multivariate Cox regression model, and the results showed that patients with ESCC with elevated CCL4 and MIF expression exhibited preferable PFS and OS outcomes (*P* = 0.028, 0.048, 0.017, 0.026, Figure 3a-d, respectively). Multivariate Cox regression analysis was conducted with age, sex, TNM stage, smoking, and expression of both CCL4 and MIF expression taken into account, and the results revealed that only MIF expression was an independent predictor of PFS and OS (*P* = 0.024 and 0.045, supplementary figure S2 a-b).

### A new prognostic model with CCL4 and MIF levels

Based on the results of our survival analyses, we propose a new cytokine prognostic model based on the expression of serum CCL4 and MIF. We designated the low-risk group as having high expression of both the cytokines, and the high-risk group as having other expressions (regardless of their identity). Our results revealed that the model significantly stratified risk (low or high) for both PFS and OS (*P* = 0.001, Figure 3e; *P* < 0.001, Figure 3g). Moreover, forest plot analysis revealed a promising predictive value regarding PFS and OS of the two-cytokine model (*P* = 0.003, Figure 3f; *P* = 0.002, Figure 3h).

### Verification of CRT prognostic capacity of CCL4, CXCL8, and MIF in tissues

**Figure 4:**
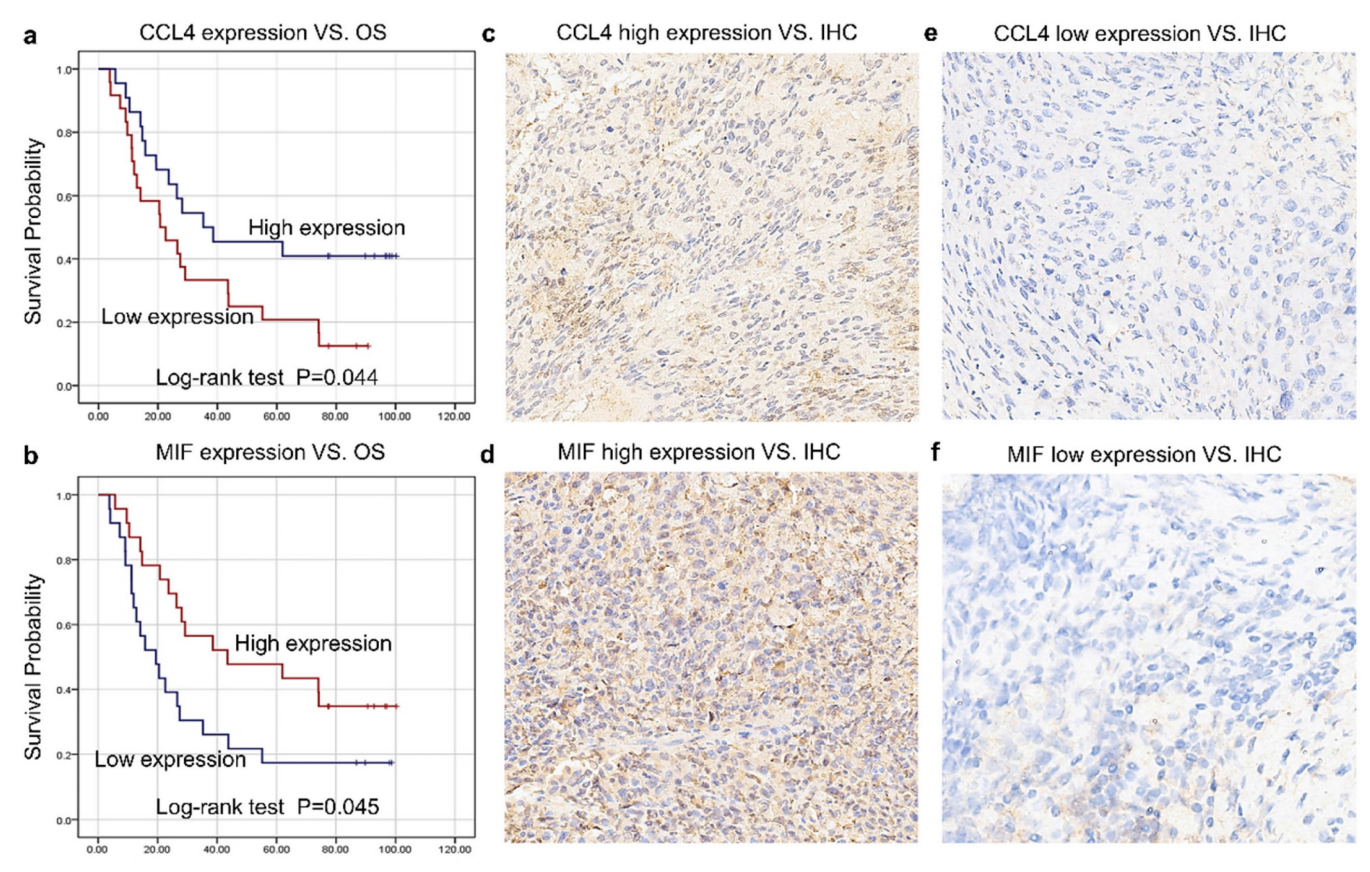
** Verification of CRT prognostic capacity of CCL4, CXCL8, and MIF in tissues.** a. Higher CCL4 level was correlated closely with greater OS (*P* = 0.044). b. Higher MIF level was positively correlated with greater OS (*P* = 0.045). c-f. The expression level of positive cells in the tumor tissues of CCL4 and MIF.

The histological expression levels of CCL4, MIF, and CXCL8 were further detected via IHC in the remaining 46 patients enrolled in this study from May 2015 to July 2018 at Tianjin Medical University Cancer Institute and Hospital. All patients were recruited in a clinical trial (NCT02959385) and received CRT with cisplatin-based chemotherapy and concomitant radiotherapy (daily dose of 2.0 Gy for a total of 40-60 Gy over 4-6 weeks). All patients were followed up until December 12, 2023. At the last follow-up, 12 patients were alive and 34 had died. Table 2 summarizes the relationships between the tissue expression levels of CCL4 and MIF and the clinicopathological features. The expression levels of CCL4-and MIF were shown in Figure 4c-f. Higher CCL4 levels were closely correlated with greater OS (*P* = 0.044; Figure 4a). Higher MIF levels were positively correlated with greater OS (*P* = 0.045, Figure 4b), which was similar to the serum results.

### Evaluation of immune cell infiltration and immune-related pathways of CCL4 and MIF

**Figure 5:**
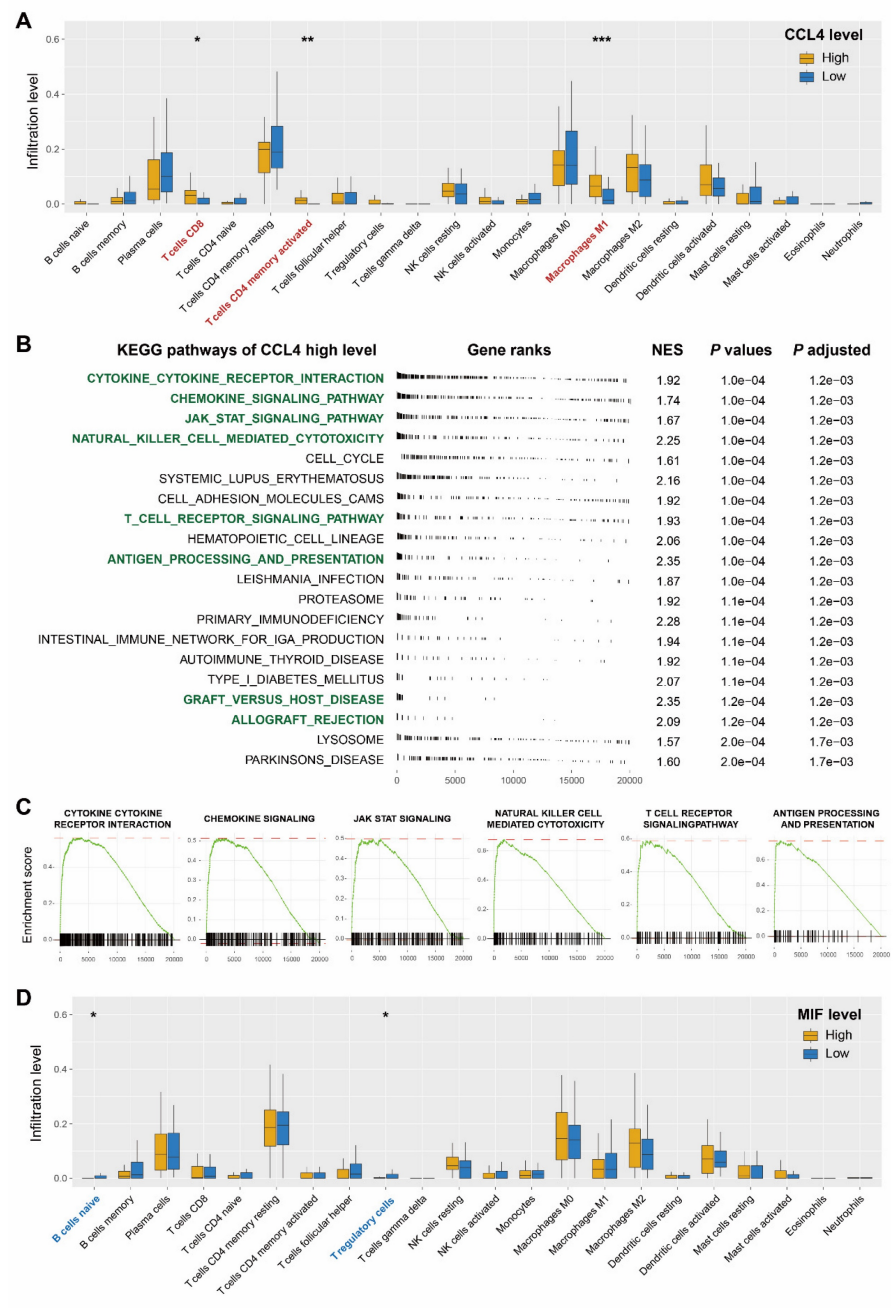
** Evaluation of immune cell infiltration and regulated immune-related pathways.** a. Correlation matrix of all 22 types of immune cell subtype compositions in CCL4-high group and -low group. b. Immune-related pathways were upregulated in the CCL4-high group. c. GSEA showed that the cytokine-cytokine-receptor-interaction signaling pathway, chemokine-signaling-pathway, jak-stat-signaling-pathway, natural-killer-cell-mediated-cytotoxicity, T-cell-receptor-signaling-pathway, and antigen-processing-and-presentation signaling pathway were significantly upregulated in CCL4-high group. The value of normalized enrichment score is displayed in the figure. d. Correlation matrix of all 22 types of immune cell subtype compositions in MIF-high group and MIF-low group. * represents p < 0.05, ** represents p < 0.01; *** represents p < 0.001.

Owing to the immunomodulatory effects of cytokines, immune cell infiltration was investigated to shed further light on the immunological regulation of CCL4 and MIF. Eighty-two patients with ESCC in the Cancer Genome Atlas database were used for statistical analysis. The boxplots showed that patients with ESCC with higher expression of CCL4 harbored an enhanced infiltration proportion of activated CD4 T memory cells, CD8 T cells, and macrophage M1 cells (all *P* < 0.05, Figure 5a). Given the above findings, we conducted KEGG GSEA to explore whether immune response-related pathways were regulated in the CCL4 high-expression subgroup. The results revealed that numerous immune-related KEGG pathways were significantly enriched, including the cytokine-cytokine receptor interaction signaling pathway, chemokine signaling pathway, JAK STAT signaling pathway, natural killer cell-mediated cytotoxicity, T cell receptor signaling pathway, antigen processing and presentation, graft versus host disease, and allograft rejection (Figure 5b, c). The boxplots showed that higher MIF expression was associated with a lower proportion of naïve B cells and T regulatory cells (Figure 5d).

## Discussion

Cytokines play an important role in tumor proliferation, angiogenesis, metastasis, immune escape, and various cytokines have been found to be statistically significantly associated with the survival of patients with ESCC 11, 12, 15. However, little is known about the relationship between cytokine profiles and ESCC CRT response. CRT is used extensively for locally advanced ESCCs, and resistance to IR is a major cause of cancer treatment failure and poor survival 16, 17. In the present prospective study, we compared the cytokine profiles of ESCC patients. Our study demonstrated that high serum levels of CCL4 and MIF were positively associated with a good CRT response and favorable outcomes in patients with ESCC. CRT is used extensively for locally advanced ESCCs, and IR resistance is a major cause of cancer treatment failure 18, 19.

CCL4, CXCL8, and MIF have received considerable attention for their roles in tumor proliferation, differentiation, angiogenesis, and tumor progression. MIF was originally identified as a product isolated from the supernatants of activated T lymphocyte cultures and was one of the first functional cytokines to be identified when it was shown to inhibit the random migration of macrophages in experiments characterizing delayed-type hypersensitivity 20, 21. MIF plays a key role in fundamental processes that control cell proliferation, differentiation, angiogenesis, and tumor progression 15,22-24. Numerous studies have demonstrated remarkable overexpression of MIF in several types of human cancer 25-29. However, these studies have shown contradictory results regarding the role of MIF in cancer progression and patient prognosis 25,27,29,30. To date, little is known about the role of MIF in CRT response in patients with ESCC. Our study showed that serum MIF was a reliable and predictive biomarker of CRT response and prognosis in patients with ESCC. In response to various stimuli, the binding of MIF to CD44/CD74 leads to sustained activation of the MAPK/ERK1 and PI3K/AKT pathways and suppression of the P53 pathway 31, 32. Tanese *et al.* demonstrated that autocrine MIF-CD74 signaling regulates the phosphorylation of AKT Ser473, resulting in an increased expression of CXCL8^33^. Therefore, the MIF-CD74 complex initiates a cascade that leads to CXCL8 transcription and secretion. CXCL8, which contributes to the progression of several types of human cancer progression 34, was also significantly higher in patients with excellent CRT response in our study. Ma *et al.* reported that high serum levels of CXCL8 are strongly correlated with clinical tumor stage and lymph node metastasis 35. A phase III clinical trial demonstrated that elevated CXCL8 levels were associated with OS irrespective of treatment in locoregionally advanced head and neck cancer; however, tirapazamine/cisplatin treatment seemed to be beneficial for patients with high CXCL8 36. Our findings showed that high CXCL8 levels in the serum predicted better CRT response and greater PFS, although the difference was not statistically significant. Our study revealed a significant correlation between MIF and CXCL8 (supplementary figure S1), which was consistent with previous studies showing that CXCL8 is downstream of MIF in certain signaling pathways.

Dumitru *et al.* demonstrated that head and neck cancer-derived MIF elicits increases inflammatory responses by inducing the release of CCL4^37^; however, the biological mechanisms of the interaction between the two cytokines have not been completely elucidated 13, 38, 39. Recent evidence suggests that increased CCL4 expression enhances the invasion and migration of prostate cancer cells 40 and promotes tumor growth and angiogenesis 41. Trellakis *et al.* demonstrated a higher concentration of CCL4 in the peripheral blood of patients with HNSCC 42. In contrast, high levels of CCL4 expression in ESCCs were correlated with a more favorable prognosis, suggesting a role in the recruitment of tumor-infiltrating CD8+ T lymphocytes and influencing the cancer microenvironment 43. Consistently, higher serum CCL4 levels were associated with better CRT response and greater PFS in the patients with ESCC enrolled in our study. Moreover, our data demonstrated that CCL4 expression was positively correlated with CXCL8 expression in the serum, and similar results were observed for CXCL8 levels and MIF. These results are consistent with those of previous studies, which showed that MIF, CXCL8, and CCL4 may be involved in the same signaling pathways and play systematic roles in ESCC progression and CRT response. In accordance with MIF and CXCL8, the clinical significance of CCL4 was not consistent, even opposite, in different cancers, suggesting that the biological functions of these cytokines in tumorigenesis and therapy response are complicated and labile and may be tumor type-specific.

Above all, majority of studies have shown that elevated CCL4 and MIF play a promoting role in cancer developments and progression. However, patients with high CCL4 and MIF may benefit from remodeling of the immune microenvironment induced by chemoradiotherapy. Recent evidence suggests that the inflammatory microenvironment is reshaped by tumor cells after radiotherapy. Ma *et al.* reported that the tumor immune microenvironment was remodeled, and anti-tumor immunity centered on B cells was observed after CRT 44. In this study, all patients received standard radiation plus cisplatin-based chemotherapy. Moreover, patients with higher expression levels of CCL4 and MIF had more infiltration of immune cells, which made it better for patients with higher levels of CCL4 and MIF who may be more sensitive to CRT, thus having longer PFS and OS. Further studies are needed to clarify the roles and mechanisms of CCL4 and MIF in the development of tumors and the regulation of the ESCC response to CRT in detail. Moreover, based on our data, we propose, for the first time, a new and simple prognostic model for patients with ESCC expressing CCL4 and MIF. Our data provide evidence that this type of cytokine prognostic model can effectively predict the CRT response in ESCC and may serve as a useful prognostic index for patients with locally advanced ESCC.

In summary, the present study highlights the clinical significance of cytokines in patients with ESCC and reveals the promising predictive value of CCL4, CXCL8, and MIF for ESCC PFS, OS, and CRT response. Our results provide a preliminary basis for the concept that high serum levels of CCL4, CXCL8, and MIF during pretreatment are associated with a better CRT response, and high serum levels of CCL4 and MIF are associated with favorable outcomes. These results were verified in the tissues. Moreover, forest plot analysis showed that CCL4, MIF, and the new cytokine prognostic model were excellent independent predictors of patient survival and chemoradiosensitivity.

## Conclusions

Our study was verified by histology, hematology and database. Our data suggest that CCL4 and MIF are promising molecular targets for developing novel combinatorial therapeutic strategies to overcome CRT resistance in ESCC. However, there were insufficient number of ESCCs in this study, and additional clinical studies and basic research with a larger sample size are needed to confirm the role of cytokines in prognostic prediction and CRT response in patients with ESCC.

## Supplementary Material

Supplementary figures.

Supplementary table.

## Figures and Tables

**Table 1:** Clinical pathological correlation of serum cytokines in ESCCs

Variable	CCL4[cases]			MIF [cases]			CXCL8[cases]		
	Low level	High level	P*	Low level	High level	P*	Low level	High level	P*
Gender			0.199			0.081			0.54
Male	22	35		26	32		28	27	
Female	5	3		1	7		5	3	
Age			0.515			0.649			0.516
<60	12	20		14	18		17	13	
≥60^†^	15	18		13	21		16	17	
Operation			0.788			0.059			0.607
Yes	7	11		4	14		8	9	
No	20	27		23	25		25	21	
T categories			0.160			0.075			0.227
T1	0	3		2	1		0	3	
T2	1	3		3	1		2	2	
T3	21	30		17	35		26	23	
T4	5	2		5	2		5	2	
N categories			0.992			0.442			0.718
N0	4	6		4	6		4	6	
N1	10	13		7	16		11	11	
N2	10	14		11	14		14	9	
N3	3	5		5	3		4	4	
TNM categories			0.547			0.068			0.551
I	0	1		0	1		0	1	
Ⅱ	3	7		5	5		4	6	
III	16	23		12	28		20	17	
IV	8	7		10	5		9	6	
Multifocal lesions			0.311			0.329			0.181
Yes	2	6		2	6		2	5	
No	25	32		25	33		31	25	
Tumor location			0.158			0.901			0.334
Upper	8	9		7	11		11	7	
Middle	12	25		16	21		16	20	
Lower	7	4		4	7		6	3	
CRT response			0.002			0.008			0.001
CR/PR	8	26		9	26		11	22	
SD/PD	19	12		18	13		22	8	

Abbreviation: ESCC, esophageal squamous cell carcinoma; T, tumor; N, node; M, metastases; CR, complete response; PR, partial response; SD, stable disease; PD, progressive disease. *Chi-square test; †Mean age

**Table 2:** Clinical pathological correlation of CCL4, MIF in ESCC tissues

Variable	CCL4[cases]			MIF [cases]		
	Low level	High level	P*	Low level	High level	P*
Gender			0.175			0.681
Male	22	17		19	20	
Female	2	5		4	3	
Age			0.136			0.555
<60	14	8		12	10	
≥60^†^	10	14		11	13	
T categories			0.317			0.112
T1	0	0		0	0	
T2	1	1		0	2	
T3	23	19		23	19	
T4	0	2		0	2	
N categories			0.827			0.523
N0	2	3		1	4	
N1	20	16		19	17	
N2	1	2		2	1	
N3	1	1		1	1	
CRT response			0.575			0.475
CR/PR	18	18		19	17	
SD/PD	6	4		4	6	

Abbreviation: ESCC, esophageal squamous cell carcinoma; T, tumor; N, node; M, metastases; CR, complete response; PR partial response; SD, stable disease; PD, progressive disease. *Chi-square test; †Mean age

## Abbreviations

ESCC: Esophageal squamous cell carcinoma
IR: Radiotherapy
CRT: Chemoradiotherapy
TCGA: The cancer genome atals
AJCC: American Joint Committee on Cancer
CR: Complete response
PR: Partial response
SD: Stable disease
PD: Progressive disease
IHC: Immunohistochemistry
GSEA: Gene set enrichment analysis
KEGG: Kyoto Encyclopedia of Genes and Genomes
ROC: Receiver operating characteristic
PFS: Progression-free survival
OS: Overall survival
