# Supplementary material for: CCL4 and MIF: Prognostic Biomarkers for Evaluating the Chemoradiotherapy Response and Prognosis in Patients with ESCC
Source: J Cancer. 2025 Mar 3;16(6):2015–25. doi: 10.7150/jca.104088 (PMC11905412; doi:10.7150/jca.104088)
Supplement: Supplementary file 1 — Supplementary figures. [file jcav16p2015s1.pdf]

Supplementary Figure Legends

Figure S1: Correlations between the expression levels of CCL4, CXCL8 and MIF.

A. Spearman's correlation coefficient analysis revealed significant positive correlation was found between the levels of CCL4 and CXCL8 ($P < 0.001$). B. No correlation was found between the levels of CCL4 and MIF ($P = 0.866$). C. A significant positive correlation was found between the levels of MIF and CXCL8 (P=0.018).

Figure S2: Multivariate Cox regression models

We further conducted multivariate Cox regression models with age, sex, TNM stage, smoking and expression of both CCL4 and MIF expression taken into account, MIF expression was independent predictor for PFS(A) and OS(B).

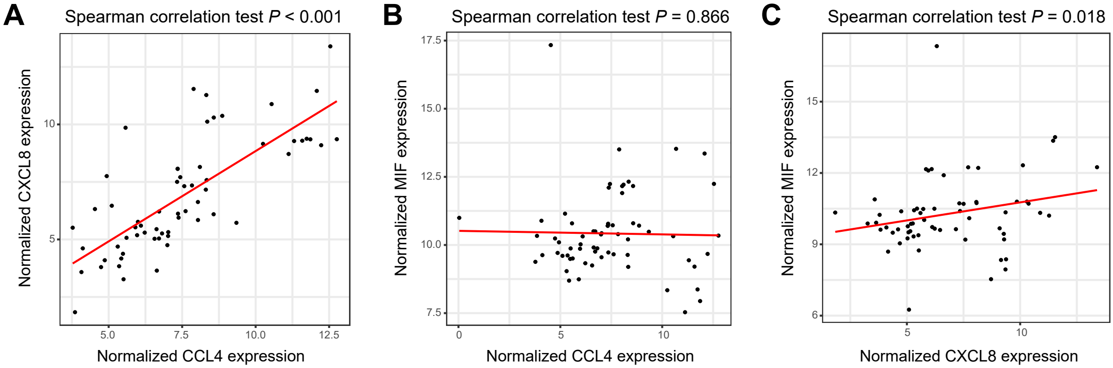

A
Spearman correlation test P < 0.001
Normalized CXCL8 expression
Normalized CCL4 expression
5
10
5.0
7.5
10.0
12.5
B
Spearman correlation test P = 0.866
Normalized MIF expression
Normalized CCL4 expression
7.5
10.0
12.5
15.0
17.5
0
5
10
C
Spearman correlation test P = 0.018
Normalized MIF expression
Normalized CXCL8 expression
6
9
12
15
5
10

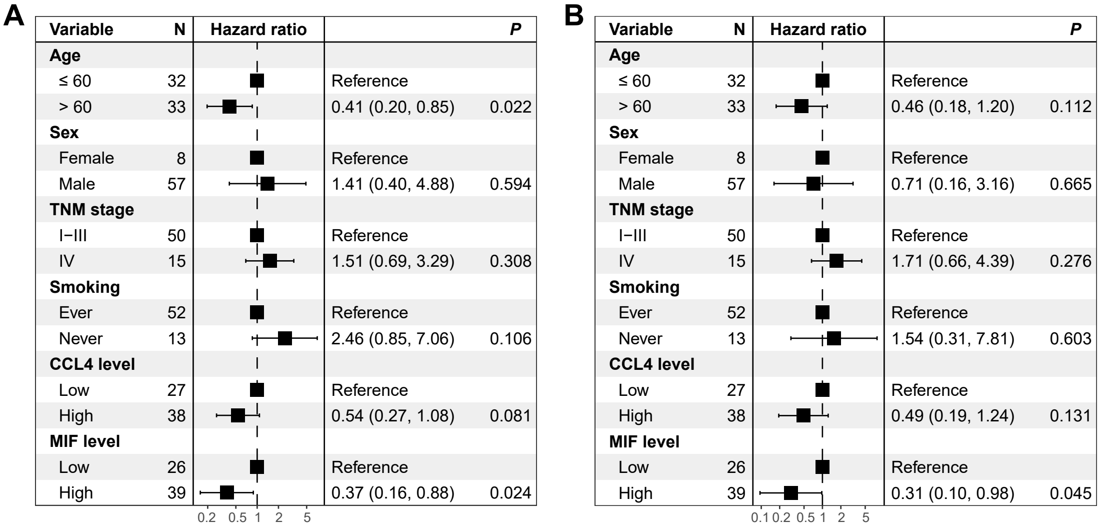
A
Variable
N
Hazard ratio
P
Age
≤ 60 32 Reference
> 60 33 0.41 (0.20, 0.85) 0.022
Sex
Female 8 Reference
Male 57 1.41 (0.40, 4.88) 0.594
TNM stage
I−III 50 Reference
IV 15 1.51 (0.69, 3.29) 0.308
Smoking
Ever 52 Reference
Never 13 2.46 (0.85, 7.06) 0.106
CCL4 level
Low 27 Reference
High 38 0.54 (0.27, 1.08) 0.081
MIF level
Low 26 Reference
High 39 0.37 (0.16, 0.88) 0.024
0.2 0.5 1 2 5
B
Variable
N
Hazard ratio
P
Age
≤ 60 32 Reference
> 60 33 0.46 (0.18, 1.20) 0.112
Sex
Female 8 Reference
Male 57 0.71 (0.16, 3.16) 0.665
TNM stage
I−III 50 Reference
IV 15 1.71 (0.66, 4.39) 0.276
Smoking
Ever 52 Reference
Never 13 1.54 (0.31, 7.81) 0.603
CCL4 level
Low 27 Reference
High 38 0.49 (0.19, 1.24) 0.131
MIF level
Low 26 Reference
High 39 0.31 (0.10, 0.98) 0.045
0.1 0.2 0.5 1 2 5
